# Autoinflammatory mutation in *NLRC4* reveals a leucine-rich repeat (LRR)–LRR oligomerization interface

**DOI:** 10.1016/j.jaci.2018.04.033

**Published:** 2018-12

**Authors:** Fiona Moghaddas, Ping Zeng, Yuxia Zhang, Heike Schützle, Sebastian Brenner, Sigrun R. Hofmann, Reinhard Berner, Yuanbo Zhao, Bingtai Lu, Xiaoyun Chen, Li Zhang, Suyun Cheng, Stefan Winkler, Kai Lehmberg, Scott W. Canna, Peter E. Czabotar, Ian P. Wicks, Dominic De Nardo, Christian M. Hedrich, Huasong Zeng, Seth L. Masters

**Affiliations:** a Inflammation Division, Walter and Eliza Hall Institute of Medical Research, Parkville, Australia; i Structural Biology Division, Walter and Eliza Hall Institute of Medical Research, Parkville, Australia; b Department of Medical Biology, University of Melbourne, Parkville, Australia; c Department of Rheumatology, Guangzhou Women and Children's Medical Centre, Guangzhou, China; d Immunology Laboratory, Guangzhou Institute of Paediatrics, Guangzhou, China; e Department of Pediatrics, University Hospital and Faculty of Medicine Carl Gustav Carus, TU Dresden, Dresden, Germany; f Department of Chemical Biology, Guizhou Medical University, Guiyang, China; g Division of Pediatric Stem Cell Transplantation and Immunology, University Medical Center Hamburg Eppendorf, Hamburg, Germany; h Pediatric Rheumatology/RK Mellon Institute, Children's Hospital of Pittsburgh of UPMC, Pittsburgh, Pa; j Rheumatology Department, Royal Melbourne Hospital, Parkville, Australia; k Department of Women's & Children's Health, Institute of Translational Medicine, University of Liverpool, Liverpool, United Kingdom; l Department of Paediatric Rheumatology, Alder Hey Children's NHS Foundation Trust Hospital, Liverpool, United Kingdom

**Keywords:** Autoinflammatory disease, periodic fever syndrome, NLRC4, macrophage activation syndrome, inflammasome, Nod-like receptor, IPAF, IL-18, AIFEC, Autoinflammation with infantile enterocolitis, ASC, Apoptosis-associated Speck-like protein containing a caspase recruitment domain, CARD, Caspase activation and recruitment domain, CRISPR, Clustered Regularly Interspaced Short Palindromic Repeats, CSF, Cerebrospinal fluid, HD, Hinge domain, IL-18BP, IL-18 binding protein, KO, Knockout, LRR, Leucine-rich repeat, MAS, Macrophage activation syndrome, NAIP, NLR family apoptosis inhibitor protein, NBD, Nucleotide-binding domain, NLRC4, NOD-like receptor family CARD-containing 4 protein, NLRC4-AID, NLRC4-associated autoinflammatory disorder, NLRP3, NOD-like receptor family, pyrin domain containing 3, NOD, Nucleotide-binding oligomerization domain, T3SS, Type 3 secretion system, WT, Wild-type

## Abstract

**Background:**

Monogenic autoinflammatory disorders are characterized by dysregulation of the innate immune system, for example by gain-of-function mutations in inflammasome-forming proteins, such as NOD-like receptor family CARD-containing 4 protein (NLRC4).

**Objective:**

Here we investigate the mechanism by which a novel mutation in the leucine-rich repeat (LRR) domain of NLRC4 (c.G1965C, p.W655C) contributes to autoinflammatory disease. Methods: We studied 2 unrelated patients with early-onset macrophage activation syndrome harboring the same *de novo* mutation in *NLRC4*. *In vitro* inflammasome complex formation was quantified by using flow cytometric analysis of apoptosis-associated speck-like protein containing a caspase recruitment domain (ASC) specks. Clustered Regularly Interspaced Short Palindromic Repeats (CRISPR)/Cas9 techniques and lentiviral transduction were used to generate THP-1 cells with either wild-type or mutant *NLRC4* cDNA. Cell death and release of IL-1β/IL-18 were quantified by using flow cytometry and ELISA, respectively.

**Results:**

The p.W655C NLRC4 mutation caused increased ASC speck formation, caspase-1–dependent cell death, and IL-1β/IL-18 production. ASC contributed to p.W655C NLRC4–mediated cytokine release but not cell death. Mutation of p.W655 activated the NLRC4 inflammasome complex by engaging with 2 interfaces on the opposing LRR domain of the oligomer. One key set of residues (p.D1010, p.D1011, p.L1012, and p.I1015) participated in LRR-LRR oligomerization when triggered by mutant NLRC4 or type 3 secretion system effector (PrgI) stimulation of the NLRC4 inflammasome complex.

**Conclusion:**

This is the first report of a mutation in the LRR domain of NLRC4 causing autoinflammatory disease. c.G1965C/p.W655C NLRC4 increased inflammasome activation *in vitro*. Data generated from various *NLRC4* mutations provides evidence that the LRR-LRR interface has an important and previously unrecognized role in oligomerization of the NLRC4 inflammasome complex.

## Graphical abstract

**Figure:**
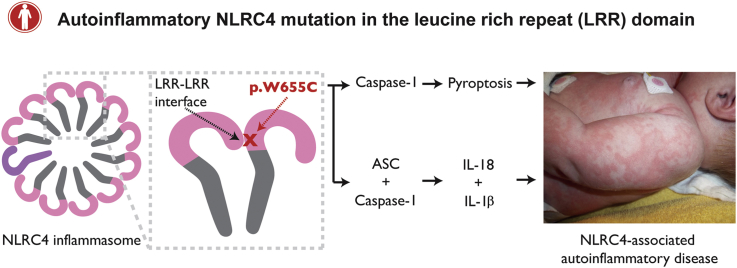

Inflammasomes are large multimeric complexes formed in response to pathogen-associated or damage-associated molecular patterns. Some innate immune sensors oligomerize with the adaptor protein apoptosis-associated speck-like protein containing a caspase recruitment domain (ASC) and caspase-1 to form a platform for the cleavage of pro–IL-1β and pro–IL-18 to their active forms. Gain-of-function mutations in inflammasome-forming proteins are a major cause of monogenic autoinflammatory disorders, a heterogeneous group of conditions characterized by innate immune dysregulation.

NOD-like receptor family caspase activation and recruitment domain (CARD)–containing 4 protein (NLRC4) forms an inflammasome in response to type 3 secretion system (T3SS) proteins from invading gram-negative bacteria, such as *Salmonella* species. Components of T3SS are recognized by cytosolic sensors known as NLR family apoptosis inhibitor proteins (NAIPs).^1^, ^2^, ^3^ NAIP proteins associate with NLRC4, initiating a conformational change that allows for NLRC4 oligomerization through self-propagation of the nucleotide-binding oligomerization domain (NOD).^4^, ^5^

Mutations in the NOD of NLRC4 result in autoinflammation, with a spectrum of clinical manifestations ranging from cold-induced urticaria to life-threatening macrophage activation syndrome (MAS) with severe enterocolitis.^6^, ^7^, ^8^, ^9^, ^10^ NLRC4-associated autoinflammatory disorders (NLRC4-AIDs) are characterized by high levels of free IL-18 in the serum of patients, distinguishing it from other monogenic inflammasomopathies, such as Familial Mediterranean Fever or Cryopyrin Associated Periodic Syndrome. Importantly, successful treatment with a recombinant IL-18 binding protein (IL-18BP) has been reported in 1 patient with autoinflammation with infantile enterocolitis (AIFEC; OMIM 616050), an NLRC4-AID.^11^

Here we identify a previously unknown mutation in the leucine-rich repeat (LRR) domain of NLRC4 in 2 unrelated patients with MAS. This is the first report of such a mutation in *NLRC4*, and we provide *in vitro* evidence of the importance of LRR-LRR interactions in the disease pathophysiology in these patients.

## Methods

### Patient and study approval

Informed consent for genetic sequencing was obtained from the patients' guardians. Patient P1 was recruited through routine care. Patient P2 and age- and sex-matched control subjects were recruited through the Guangzhou Women and Children's Medical Center Ethics Committee (2016021602). Further informed consent was obtained for publication of case descriptions and clinical images.

### Genetic analysis

Genomic DNA was extracted from whole blood using the QIAamp DNA Micro Kit (56304; Qiagen, Hilden, Germany). Targeted sequencing was performed on patient P1. *NLRC4* was amplified by means of PCR and sequenced using the Sanger method and primers listed in Table E1 in this article's Online Repository at www.jacionline.org. Whole-exome sequencing was performed on patient P2 and patient P2's family members using the Agilent SureSelect Human All Exon V6 kit (Agilent Technologies, Santa Clara, Calif) sequenced on an Illumina platform (Illumina, San Diego, Calif). Bioinformatics analysis with read mapping and variant calling was performed using the Genome Analysis Toolkit Haplotype Caller. The variant of interest was confirmed with Sanger sequencing.

### Serum cytokine analysis

For patient P1, serum was diluted in sample buffer and assayed in multiplex on a Luminex Magpix system (Bio-Rad Laboratories, Hercules, Calif). Human IL-18BPa beads were generated with magnetic beads (Bio-Rad Laboratories) conjugated to clone MAB1192 and detected with clone BAF119 (both from R&D Systems, Minneapolis, Minn). Bioplex Pro group II cytokine standard was used for IL-18, whereas recombinant human IL-18BPa–Fc (R&D Systems) was used for IL-18BP. Patient P2's serum cytokine levels were quantified by using an ELISA for IL-1β (CHE001; 4A Biotech, Beijing, China) and IL-18 (CHE007; 4A Biotech), according to the manufacturer's guidelines.

### Generation of NLRC4-deficient cells

The method of generating knockout (KO) cells using Clustered Regularly Interspaced Short Palindromic Repeats (CRISPR)/Cas9 techniques, as well as lentivirus production, has been previously described.^12^, ^13^ The single guide RNA constructs used to make *CASPASE1* KO, *CASPASE8* KO, and *PYCARD* KO cells have been previously described.^14^, ^15^, ^16^ Genetic deletion of *NLRC4* was achieved using single guide RNA oligonucleotides targeting exon 2 (see Table E1).

### Generation of lentiviral constructs

Lentiviral constructs were generated by means of amplification of *NLRC4* cDNA with Phusion DNA polymerase (M0530S; New England BioLabs, Ipswich, Mass) using primers flanked by restriction enzyme sequences, which allowed for cloning into the pFUGW backbone (see Table E1).^17^ Both pFUGW and amplified cDNA were digested with *Age*I-HF (R3552) and *BamH*1-HF restriction enzymes (R3136; New England BioLabs), followed by agarose gel electrophoresis and DNA extraction. The vector and insert were ligated with T4 DNA Ligase (B0202S; New England BioLabs).

### Site-directed mutagenesis

Site-directed mutagenesis was performed with the QuickChange Lightning Kit (210519-5; Agilent Technologies), according to the manufacturer's instructions. Mutations were introduced to constructs by using the oligonucleotides listed in Table E1.

### Cell-culture procedures

Human THP-1 and HEK293T cells were grown at 37°C in a humidified atmosphere of air with 10% CO_2_. THP-1 cells were maintained in HT RPMI (1% [wt/vol] RPMI-1640, 0.2% [wt/vol] NaHCO_3_, 0.011% [wt/vol] C_3_H_3_NaO_3_, 0.1% [wt/vol] streptomycin, and 100 U/mL penicillin) supplemented with 10% (vol/vol) FBS (Sigma-Aldrich, St Louis, Mo). HEK293T cells were maintained in Dulbecco modified Eagle medium (1% [wt/vol] D-glucose, 0.11% [wt/vol] sodium pyruvate, 0.1% [wt/vol] streptomycin, and 100 U/mL penicillin) supplemented with 10% (vol/vol) FBS (Sigma-Aldrich).

### Transduction of KO cell lines

*NLRC4* KO cells were reconstituted using third-generation lentiviral vector transduction. We were not able to generate stable cell lines carrying pathogenic *NLRC4* mutations because of high levels of spontaneous cell death. As a result, lentiviral transduction was undertaken before each experiment, as previously described.^18^ THP-1 cells, 2 × 10^6^, were infected per condition, with 1 × 10^6^ cells per well in a 6-well plate. One milliliter of viral supernatant was added to each well and supplemented with 2.5 mL of RPMI and 8 μg/mL polybrene, followed by centrifugation for 3 hours at 840*g* at 32°C. Cells were incubated overnight at 37°C, and the following day, they were washed in PBS and reseeded in fresh complete RPMI. After a further 24 hours, cells were seeded for experiments, and protein expression was determined on whole-cell lysates.

### Cell stimulation

THP-1 monocytes underwent retroviral transduction with pMXsIG_PrgI_GFP.^19^ Briefly, 3 × 10^6^ HEK293T cells were seeded in 10-cm Petri dishes. After adherence, cells were transfected with Lipofectamine 2000 (Life Technologies, Grand Island, NY) and pMXsIG_PrgI_GFP (10 μg) along with pGag-pol (5 μg) and pVSV-G (500 ng). After a media change to complete RPMI plus 10% FBS at 24 hours, viral supernatants were collected at 48 hours and stored at −80°C until required. THP-1 cells were plated in 96-well plates to a final density of 5 × 10^4^ cells per well. Priming was performed with the synthetic Toll-like receptor 2/1 agonist Pam3CSK4 (InvivoGen, San Diego, Calif) at a final concentration of 100 ng/mL for 3 hours. A titration of viral supernatant was used, with RPMI added to bring the volume of each well to 100 μL. Polybrene was added to a final concentration of 8 μg/mL. Supernatants were collected at 24 hours for cytokine quantification by means of ELISA, and cells were stained with propidium iodide (1 μg/mL; Sigma-Aldrich) to quantify cell death by using flow cytometry. NOD-like receptor family, pyrin domain containing 3 (NLRP3) was activated by treating cells with nigericin (10 μmol/L; InvivoGen) for 1 hour before collection of supernatant and cell death analysis. Where indicated, the NLRP3 inhibitor MCC950 (20 ng/mL) was used 30 minutes before stimulation with PrgI or nigericin or, in the case of mutant cell lines, after priming.

### Cytokine quantification from cell-culture supernatants

The presence of cytokines in supernatants was assessed by means of ELISA for IL-1β and IL-18 using DY201 and DY008 kits, respectively (R&D Systems), according to the manufacturer's guidelines.

### Western blot analysis

THP-1 and HEK293T cells were lysed with RIPA buffer supplemented with cOmplete protease inhibitors (11697498001; Roche Biochemicals, Mannheim, Germany). Whole-cell lysates were incubated on ice for 30 minutes and clarified by means of centrifugation. Whole-cell lysates were eluted with SDS-PAGE sample buffer, resolved on Novex 4-12% SDS-PAGE gels with MES running buffer, and subsequently transferred onto nitrocellulose membranes. Membranes were blocked overnight in 3% BSA plus 0.1% Tris-buffered saline–Tween 20 at room temperature for 1 hour and then probed overnight at 4°C with primary antibodies, including α-NLRC4 (rabbit α-NLRC4; D5Y8E; Cell Signaling Technology, Danvers, Mass), α–caspase-1 (mouse α–caspase-1 p20; AdipoGen, San Diego, Calif), α-ASC (rabbit α-ASC; sc-22514; Santa Cruz Biotechnology, Dallas, Tex), α–caspase-8 (mouse α–caspase-8; #9746; Cell Signaling Technology), and α-actin (goat α-actin; sc-1616; Santa Cruz Biotechnology).

### Time-of-flight inflammasome evaluation

Flow cytometry for quantification of ASC speck formation by time-of-flight inflammasome evaluation (TOFIE) was used as a surrogate marker of inflammasome activation.^20^ HEK293T cells were transfected with 5 ng of GFP-ASC and 10 ng of pCR3_NLRC4_VSV. ASC speck formation was quantified using flow cytometry 16 hours after transfection. For examination of the response to T3SS proteins, TOFIE was conducted in HEK293T cells stably expressing ASC-RFP through retroviral transduction.^21^ These cells were transfected with 10 ng of pCR3_NLRC4_VSV, as well as variable amounts of pMXsIG_PrgI_GFP and pCS2_hNAIP_myc, as described in the relevant figure legends.^22^

### Structural analysis

Previously published Protein Data Bank files 4KXF,^23^ 3JBL,^4^ and 6B5B^24^ were used to generate ribbon figures of NLRC4's structure in the PyMOL Molecular Graphics System (Version 2.0; Schrödinger, New York, NY). The NLRC4 active and inactive conformations were compared by aligning the LRR domains.

### Statistical analysis

Prism software (GraphPad Software, La Jolla, Calif) was used to perform 2-tailed *t* tests. Data were pooled from at least 3 independent experiments and represented as means ± SEMs, unless otherwise specified.

## Results

### Case 1

**Fig 1:**
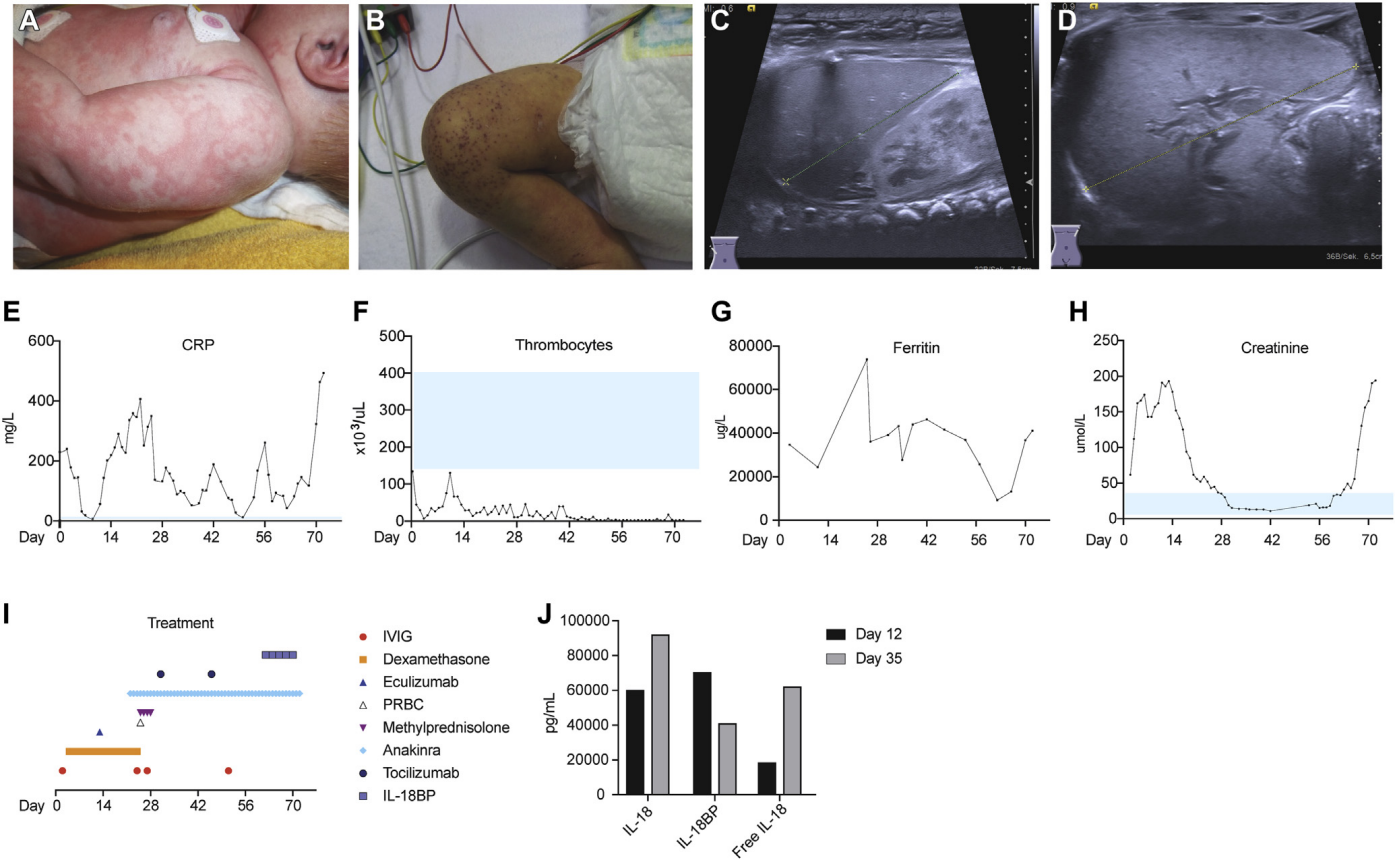
Clinical manifestations and biochemical analysis of patient P1. **A** and **B,** Dermatologic signs evolved from the initial presentation of urticaria-like rash (Fig 1, *A*) to petechiae and ecchymosis (Fig 1, *B*). **C** and **D,** Ultrasonographic images indicate hepatomegaly (Fig 1, *C*) and splenomegaly (Fig 1, *D*). **E** and **F,** Increased C-reactive protein *(CRP)* levels (Fig 1, *E*) and platelet counts (Fig 1, *F*) were improved transiently with intravenous immunoglobulin and dexamethasone. **G** and **I,** Ferritin levels (Fig 1, *G*) remained markedly increased despite treatment with numerous immunomodulatory agents (Fig 1, *I*). **H,** Acute renal injury and response to peritoneal dialysis monitored with serum creatinine levels. **J,** Serum IL-18, IL-18BP, and free IL-18 levels were assessed on days 12 and 35. *PRBC*, Packed red blood cells.

**Fig E1:**
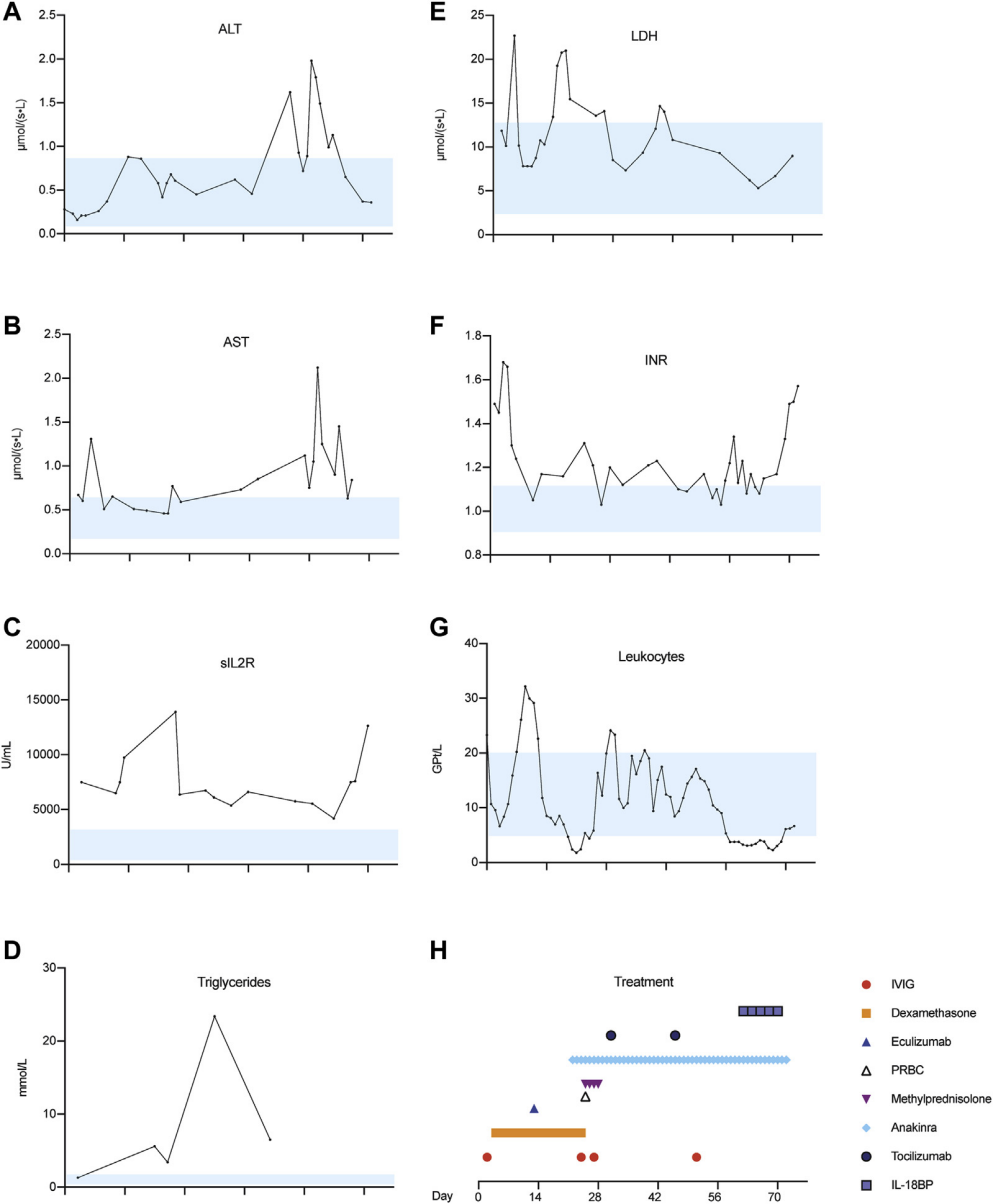
Biochemical and hematologic profile of patient P1. **A-G,** Alanine aminotransferase (*ALT*; Fig E1, *A*), aspartate aminotransferase (*AST*; Fig E1, *B*), soluble IL-2 receptor (*sIL-2R*; Fig E1, *C*), triglycerides (Fig E1, *D*), lactate dehydrogenase (*LDH*; Fig E1, *E*), international normalized ratio (*INR*; Fig E1, *F*), and leukocyte count (Fig E1, *G*) displayed over time. **H,** Treatment over the same time period. *IVIG*, Intravenous immunoglobulin; *PRBC*, packed red blood cell.

Patient P1 presented at age 11 days with high-grade fever, urticaria-like rash (Fig 1, *A*), and increased acute-phase reactant C-reactive protein levels (Fig 1, *E*). Broad-spectrum antibiotic therapy was initiated for suspected neonatal sepsis. Multiple blood and cerebrospinal fluid (CSF) cultures remained sterile. Despite antimicrobial therapy, the patient continued to deteriorate, with development of thrombocytopenia and acute renal injury (Fig 1, *F* and *H*) necessitating intermittent peritoneal dialysis. The patient had hepatosplenomegaly, and the urticaria-like rash evolved to a combination of petechiae and ecchymosis (Fig 1, *B-D*). Progressive pancytopenia, as well as an increase in soluble IL-2 receptor, ferritin, transaminases, and triglyceride levels (Fig 1, *G*, and see Fig E1 in this article's Online Repository at www.jacionline.org) provided biochemical evidence of MAS, which was confirmed on bone marrow biopsy. Multiple anti-inflammatory and immunomodulatory agents were used, including high-dose corticosteroids and the terminal complement component inhibitor eculizumab (300-mg single dose) without a significant effect (Fig 1, *I*). The patient had severe secretory diarrhea despite therapy, prompting consideration of the diagnosis of AIFEC. The IL-1 receptor antagonist anakinra (10 mg/d increased to 20 mg/d after 18 days) was used, again without clinical response, and a dose of tocilizumab (40 mg) was given, although with only short-term improvement.

The clinical situation deteriorated, with development of diffuse mucosal hemorrhage complicated by bladder clots and obstructive renal failure requiring surgical decompression. Eight weeks after presentation and after quantification of free IL-18 in the serum, a trial of recombinant IL-18BP was initiated at 2 mg/kg subcutaneously every 48 hours (Fig 1, *J*). Although there was slight improvement in diarrhea after 5 doses, thrombocytopenia persisted, and inflammatory markers remained increased. Based on the severity of symptoms, end-organ damage, and family wishes, active care was withdrawn, and the patient died at age 11 weeks, 9 weeks after admission.

### Case 2

**Fig 2:**
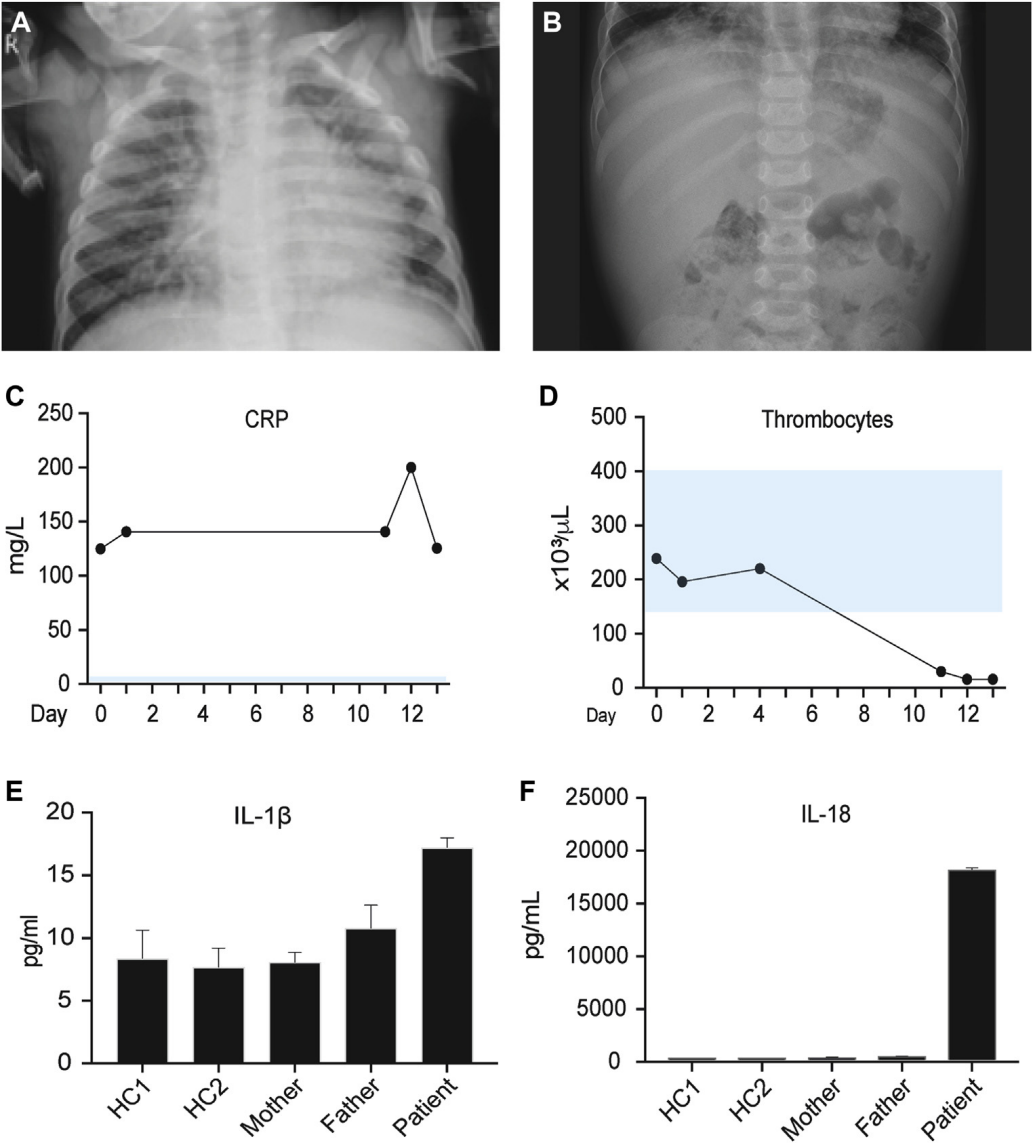
Clinical manifestations and biochemical analysis of patient P2. **A,** Chest radiograph documenting bronchopneumonia. **B,** Abdominal radiograph demonstrating hepatomegaly. **C** and **D,** C-reactive protein *(CRP)* levels (Fig 2, *C*) remained increased throughout admission with progressive thrombocytopenia (Fig 2, *D*). **E** and **F,** Serum IL-1β (Fig 2, *E*) and IL-18 (Fig 2, *F*) from healthy control subjects *(HC)*, parents, and the patient.

Patient P2 presented at age 18 months with persistent fever despite treatment for bronchopneumonia with oral cephalosporin. The patient had a history of neonatal sepsis diagnosed at day 3 of life, 2 episodes of bronchopneumonia, and intestinal intussusception requiring surgical intervention at 11 months of age. In the 6 days before this admission, patient P2 experienced cough, dyspnea, wheezing, diarrhea, abdominal pain, and maculopapular skin rash. On admission, the patient was febrile at 38.8°C, with examination revealing symmetric breath sounds with transmitted upper airway sounds and hepatomegaly. Chest radiography confirmed bronchopneumonia, and abdominal radiography revealed an enlarged liver (Fig 2, *A* and *B*). C-reactive protein levels were markedly increased (Fig 2, *C*), prompting commencement of treatment with intravenous ceftriaxone.

Given the history of recurrent infections, primary immunodeficiency was suspected, and intravenous immunoglobulin treatment was initiated. Evaluation for primary immunodeficiency revealed increased IgE levels, and the diagnosis of hyper-IgE syndrome was suspected (see Table E2 in this article's Online Repository at www.jacionline.org). Persistent fever and altered conscious state prompted consideration of central nervous system infection. CSF culture grew *Sphingomonas paucimobilis*, and targeted antimicrobial treatment was commenced. Despite this, patient P2 remained febrile, with deterioration of conscious state, progressive hepatosplenomegaly, lymphadenopathy, and thrombocytopenia (Fig 2, *D*). Subsequent CSF cultures remained sterile. The serum ferritin level at this time was markedly increased at 16,500 μg/mL (normal range, 7-140 μg/mL), as were transaminase and triglyceride levels, prompting a diagnosis of MAS. Treatment with methylprednisolone at 2.5 mg/kg/d was commenced; however, the patient died 16 days after admission. Retrospective analysis of serum from day 15 showed patient P2 had markedly increased total IL-18 levels (Fig 2, *E* and *F*).

### Genetic analysis

**Fig 3:**
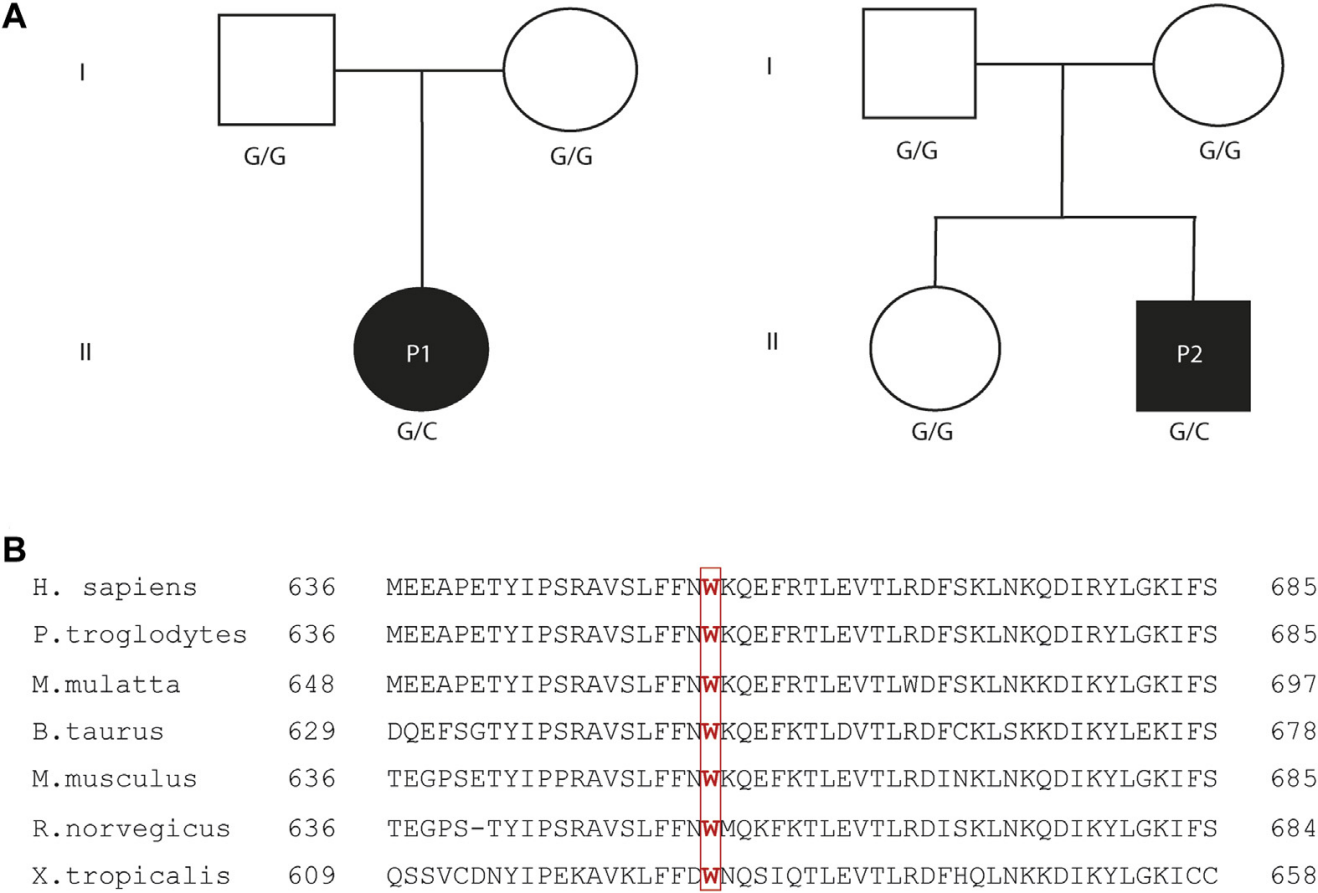
Pedigree of families. **A,** Patients P1 and P2 both carried a G>C transition at c.1965 *NLRC4* encoding a tryptophan-to-cysteine substitution at p.W655. *Solid symbols* represent affected subjects, and *open symbols* represent unaffected subjects. *Squares*, Male subjects; *circles*, female subjects. **B,** Sequence alignment across species shows that this is a highly conserved locus.

Patient P1 underwent targeted Sanger sequencing of *NLRC4* because of clinical suspicion of AIFEC. Sequencing revealed heterozygous *NLRC4* c.1965G>C transition encoding for the p.W655C variant. Patient P2 had the same variant detected by whole-exome sequencing, which was subsequently confirmed using Sanger sequencing. The sequence of *STAT3* in patient P2 was specifically reviewed, and no mutations were identified. No immediate family members of either patient had this substitution, suggesting *de novo* mutations (Fig 3, *A*). NLRC4 p.W655 is highly conserved across species (Fig 3, *B*), and p.W655C has not been documented in the Genome Aggregation Database (gnomAD).^25^ Although predicted to be benign (PolyPhen-2) or tolerated (SIFT), the suspicion of pathogenicity was such that further evaluation ensued.^26^, ^27^

### p.W655 is located in the LRR domain of NLRC4

**Fig 4:**
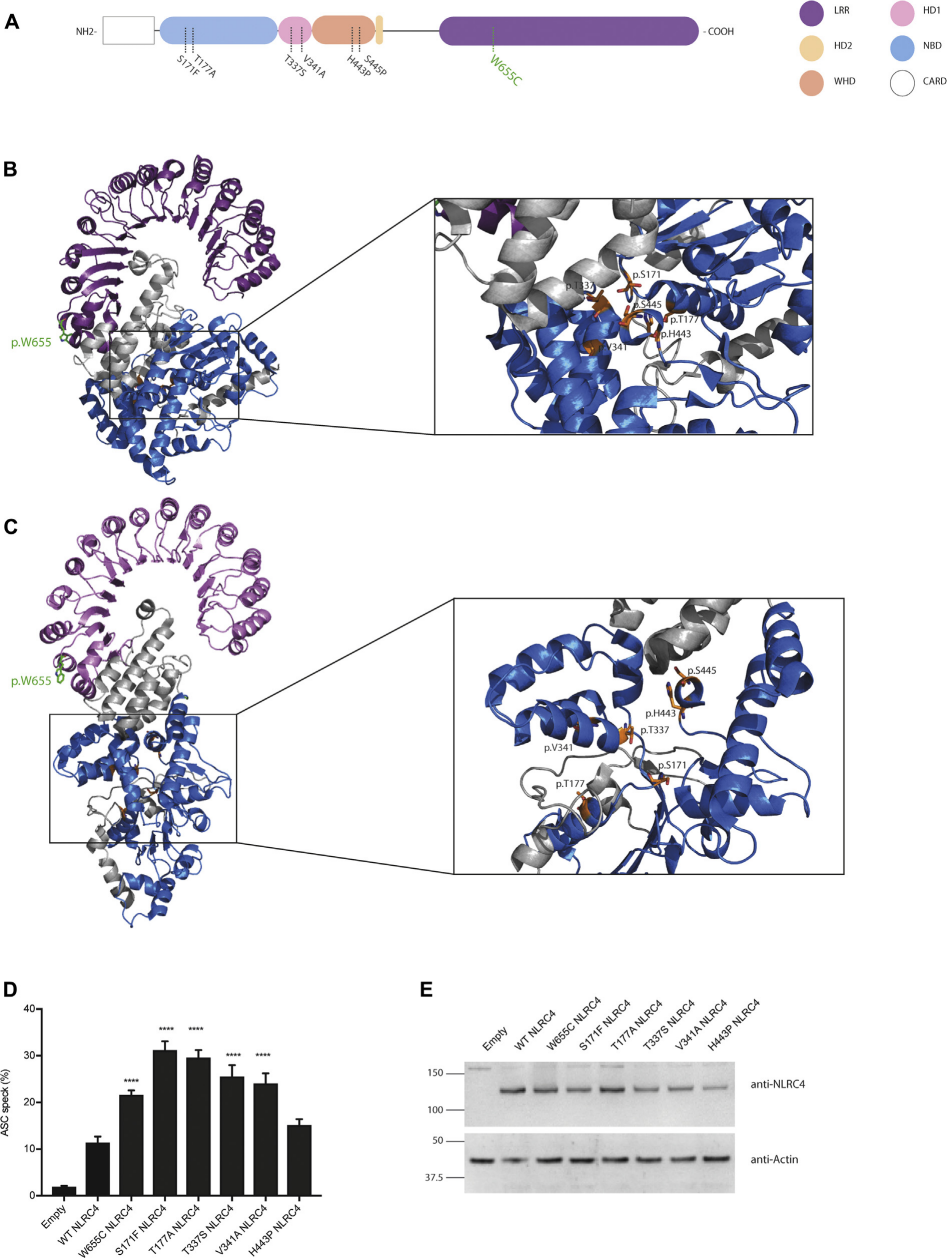
p.W655 is located in the LRR domain of NLRC4. **A,** Schematic representation of NLRC4 domains, with the variant of interest shown in green. **B** and **C,** Ribbon representation of secondary structure of autoinhibited (PDB Code 4KXF^23^; Fig 4, *B*) and active (PDB Code 6B5B^24^; Fig 4, C) NLRC4. p.W655C NLRC4 is displayed in stick format in green. **D** and **E,** ASC speck quantification of WT and NLRC4 mutants by using flow cytometry (Fig 4, *D*), with NLRC4 expression in whole-cell lysate assessed by using Western blotting (Fig 4, *E*). Data are pooled from 5 independent experiments: *P < .05, **P < .01, ***P < .001, and ****P < .0001. *NBD*, Nucleotide binding domain; *WHD*, winged helix domain.

NLRC4 consists of an N-terminal CARD, a nucleotide-binding domain (NBD), 2 hinge domains (HDs; HD1 and HD2), a winged helix domain (WHD), and a C-terminal LRR domain (Fig 4, *A*). The structure of murine NLRC4, which shares 75% sequence identity with human NLRC4, suggests that NLRC4 exists in an ADP-dependent autoinhibited monomeric conformation, with the C-terminal LRR domain occluding the central NBD (Fig 4, *B*).^23^ NLRC4 activation results in a conformational change that exposes the NBD (Fig 4, *C*).^24^ p.W655C resides in the LRR distal to the currently known mutations, which are all located around the ADP-binding regions (Fig 4, *A-C*).^6^, ^7^, ^8^, ^9^, ^10^, ^28^, ^29^

### p.W655C NLRC4 results in increased ASC speck formation

A hallmark of inflammasome activation within a single cell is formation of the so-called ASC speck. We performed experiments to determine whether expression of p.W655C NLRC4 resulted in increased ASC speck formation compared with wild-type (WT) NLRC4. Flow cytometry was used to quantify ASC speck formation as a marker for inflammasome assembly and activation. HEK293T cells were transiently transfected with ASC-GFP and various mutant forms of NLRC4. We observed significantly increased ASC speck formation in cells transfected with p.W655C NLRC4 compared with WT NLRC4. The same was also true for the other known NLRC4 mutants, except p.H443P, when expressed at similar levels (Fig 4, *D* and *E*). These data indicate that p.W655C increases inflammasome assembly.

### p.W655C NLRC4 causes increased IL-1β and IL-18 release and pyroptosis

**Fig 5:**
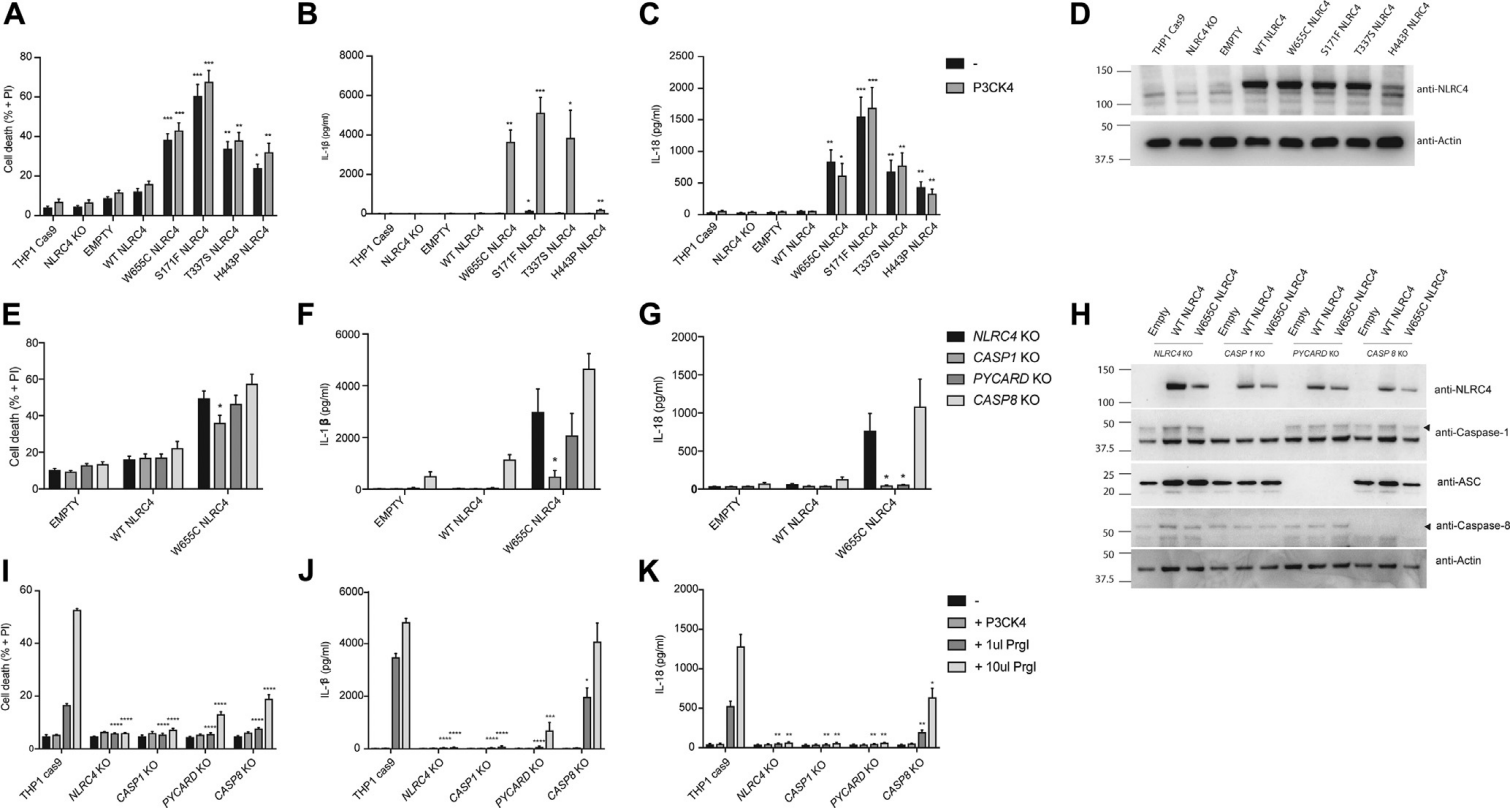
Effects of p.W655C NLRC4 on cytokine production and cell death. *NLRC4* KO THP-1 monocytes were transduced with lentivirus coding for WT or various *NLRC4* mutations (as indicated). Twenty-four hours after transduction, cells were plated and treated with Pam3CSK4. **A-C,** After 24 hours, propidium iodine *(PI)* staining was assessed by using flow cytometry to quantify cell death (Fig 5, *A*), and IL-1β (Fig 5, *B*) and IL-18 (Fig 5, *C*) secretion was measured by means of ELISA. **D,** Expression of *NLRC4* under each condition was qualified by means of Western blotting of whole-cell lysates. **E-H,***CASPASE1*, *PYCARD*, or *CASPASE8* KO THP-1 monocytes were transduced with WT or p.W655C NLRC4, and assessment of cell death (Fig 5, *E*) and IL-1β (Fig 5, *F*) and IL-18 (Fig 5, *G*) secretion was undertaken after treatment with Pam3CSK4, with expression of NLRC4 determined by using Western blotting (Fig 5, *H*). THP-1 cells, along with *NLRC4*, *CASPASE1*, *PYCARD*, or *CASPASE8* KO THP-1 monocytes, were primed for 3 hours with Pam3CSK4 and infected with 2 amounts of retrovirus expressing PrgI needle protein. **I-K,** After 24 hours, cell death (Fig 5, *I*) and IL-1β (Fig 5, *J*) and IL-18 (Fig 5, *K*) secretion were assessed with flow cytometry and ELISA, respectively. Data were pooled from at least 3 independent experiments. *P < .05, **P < .01, and ***P < .001.

**Fig E2:**
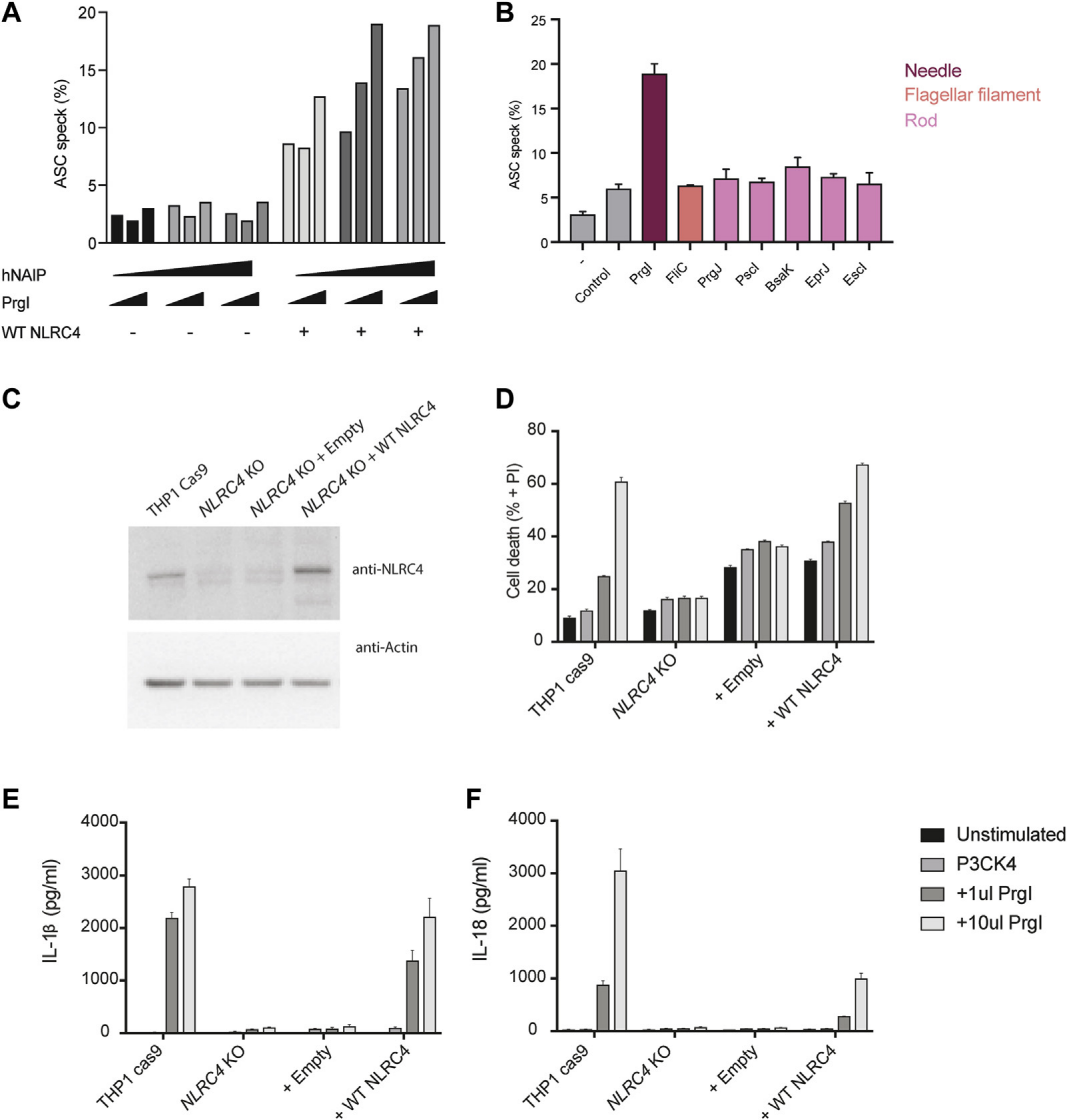
T3SS selection and validation of *NLRC4* KO THP-1 cells. HEK293T cells were transfected with WT NLRC4, as well as increasing amounts of human NAIP *(hNAIP)* and the T3SS needle protein PrgI. **A,** Flow cytometric analysis of ASC speck formation shows that WT NLRC4 is needed for ASC speck formation and that hNAIP is required for ASC specks to increase in response to low amounts of PrgI. **B,** Various T3SS proteins were transfected into HEK293T cells in addition to WT NLRC4 and hNAIP to ensure specificity of response. The ASC speck response is specific to the only needle protein tested, PrgI. **C,** THP-1–Cas9 cells infected with inducible single guide RNA targeting exon 2 of *NLRC4* were treated with doxycycline for 72 hours and expression of NLRC4 assessed with Western blotting performed on whole-cell lysates after 48 hours of rest. Cells were then primed with Pam3CSK4 and infected with 2 amounts of retrovirus expressing PrgI needle protein. **D-F,** After 24 hours of assessment of cell death (Fig E2, *D*), IL-1β (Fig E2, *E*) and IL-18 (Fig E2, *F*) secretion was undertaken.

*NLRC4*-deficienct THP-1 monocyte–like cells were generated by using CRISPR/Cas9 gene editing techniques to model p.W655C NLRC4 in a relevant human cell line (see Fig E2 in this article's Online Repository at www.jacionline.org). *NLRC4* KO THP-1 cells were transduced with lentiviral constructs carrying *NLRC4* cDNA with various mutations. THP-1 cells transiently transduced with mutant NLRC4 exhibited increased cell death and released more IL-1β and IL-18 compared with WT NLRC4 (Fig 5, *A-C*). Cell death and cytokine response were similar in p.W655C NLRC4 compared with other known disease-causing mutations. p.H443P NLRC4 was expressed at lower levels and released significantly less IL-1β than the other mutations (Fig 5, *D*). Caspase-1 deletion significantly reduced IL-1β and IL-18 secretion and decreased cell death (Fig 5, *E-G*).

### Increased IL-18 secretion, but not cell death, is dependent on ASC

The requirement of ASC in patients with NLRC4-AIDs was addressed by Romberg et al^10^ using a HEK293T overexpression system, and they determined that ASC was required for mutant NLRC4-associated caspase-1 cleavage. To further explore the requirement of ASC in a monocytic cell line, we transduced *PYCARD* KO THP-1 cells with WT or mutant NLRC4. Cell death seen with p.W655C NLRC4 was not decreased significantly by deletion of ASC, but IL-18 was markedly reduced to levels similar to those seen in *CASPASE1* KO cells (Fig 5, *E-G*). There was also a trend toward reduced IL-1β secretion. This suggests that the increased release of IL-18 (and potentially IL-1β) associated with this mutant, but not pyroptosis, is dependent on ASC. Thus cytokine release and cell death might either depend on distinct and individual pathways or require different thresholds of caspase-1 activity.

### Increased IL-1β and IL-18 secretion and cell death are not dependent on caspase-8 or NLRP3

**Fig E3:**
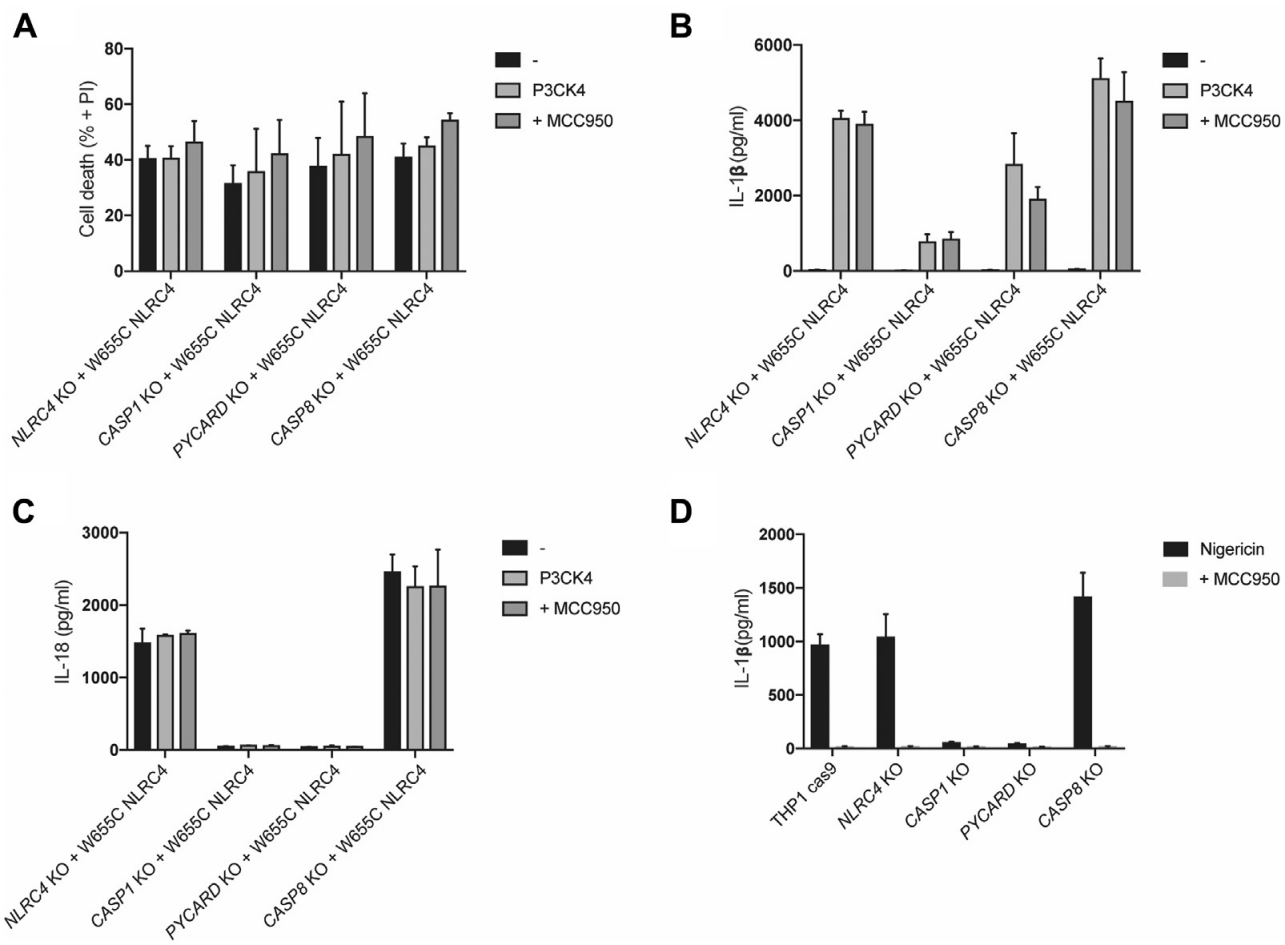
Role of NLRP3 in p.W655C NLRC4−associated inflammation. *NLRC4* KO THP-1 cells were transduced lentivirally with WT or p.W655C NLRC4. **A-C,** Contribution of NLRP3 to cell death and cytokine response was determined by using MCC950 at 20 ng/mL with cell death (Fig E3, *A*), and IL-1β (Fig E3, *B*) and IL-18 (Fig E3, *C*) secretion was assessed after 24 hours of treatment. **D,** IL-1β response to nigericin (10 μmol/L) for 1 hour with and without MCC950 pretreatment in cell lines used. Data were pooled from at least 3 independent experiments.

Caspase-8 is a downstream effector of NLRC4-induced cytokine response in the context of *Salmonella typhimurium* infection in a murine model.^30^ When transduced with NLRC4 constructs, *CASPASE8* KO THP-1 cells exhibit increased rather than abrogated cell death and IL-1β and IL-18 secretion (Fig 5, *E-H*), indicating that caspase-8 does not contribute to the increased inflammatory response but might rather have a regulatory role. Stimulation of *CASPASE8* KO cells with nigericin, an activator of NLRP3, also resulted in increased IL-1β release when compared with THP-1–Cas9 control cells (see Fig E3, *D*, in this article's Online Repository at www.jacionline.org), suggesting that the increase is not specific to NLRC4. When THP-1 cells were stimulated with PrgI, a T3SS protein, ASC contributed to cell death and cytokine response and caspase-8 contributed to cell death and IL-18 response, suggesting that the mechanism of activation might differ between pathogenic NLRC4 mutations and the physiologic response to T3SS proteins (Fig 5, *I-J*).

The presence of NLRP3 and NLRC4 in a single inflammasome complex has been reported in the setting of *S typhimurium* infection.^31^, ^32^ Therefore we explored the potential contribution of NLRP3 to p.W655C NLRC4–associated inflammation by treating cells with the specific small-molecule NLRP3 inhibitor MCC950.^33^ Cell death and IL-1β and IL-18 release were unchanged in response to coculture with MCC950 (see Fig E3). Because MCC950 completely blocked NLRP3 activation by nigericin, the presented data indicate that NLRP3 does not play a significant role in the autoinflammation seen in association with p.W655C NLRC4.

### p.W655 does not tolerate substitution

**Fig E4:**
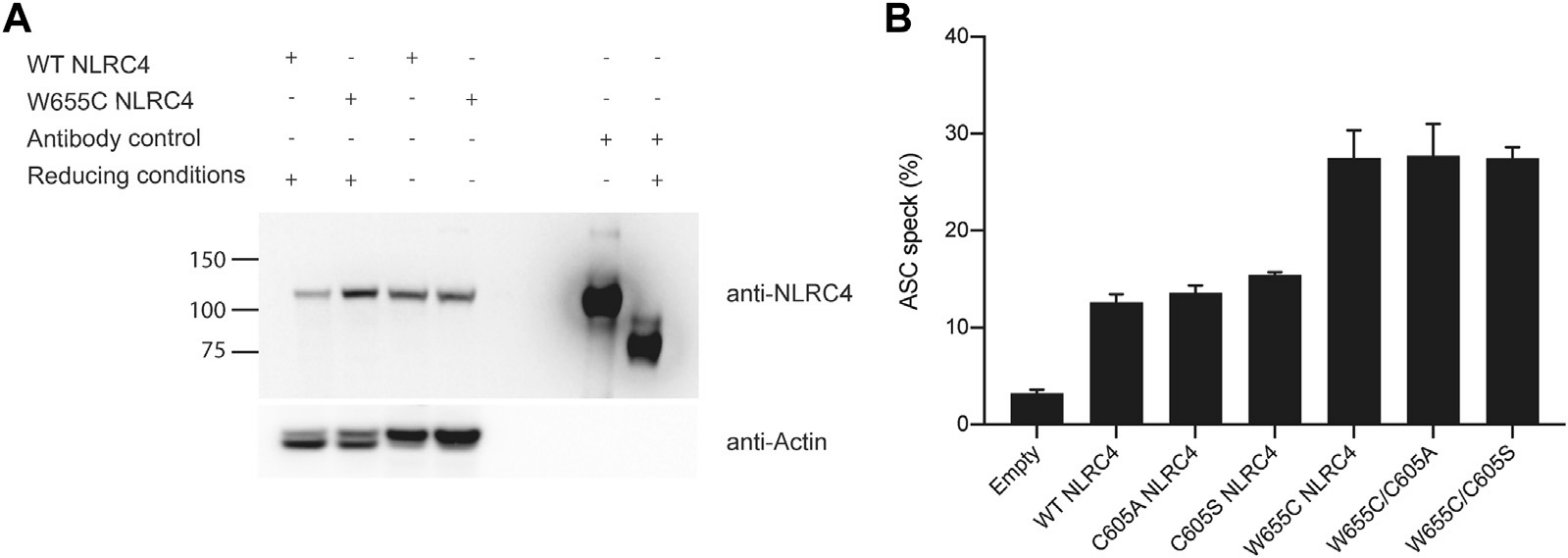
Investigation of potential disulfide bonds. **A,** HEK293T cells were transfected with WT NLRC4 or p.W655C NLRC4. After 24 hours, cells were lysed in either reducing (presence of dithiothreitol *[DTT]*) or nonreducing conditions. Protein size was compared between conditions. An antibody-only control was used to ensure that a band could be detected in both conditions. Data are representative of 3 independent experiments. **B,** ASC speck quantification using flow cytometry of WT and p.W655C NLRC4 with and without manipulation of p.C605 NLRC4. Data were pooled from 3 independent experiments.

To investigate the potential mechanisms of increased NLRC4 activation, we considered formation of a disulfide link, given that cysteine contains a sulfhydryl group that, when oxidized, might create a disulfide bond. Western blots performed in reducing or nonreducing conditions did not change the size of NLRC4, indicating that the molecule does not exist in different conformations because of a new disulfide bond (see Fig E4, *A*, in this article's Online Repository at www.jacionline.org). This was further explored through manipulation of the cysteine residue at position p.605 because this was considered to be the most likely residue with which p.W655C can form a disulfide bond. Mutation of p.C605 to alanine or serine did not change the levels of ASC speck formation (see Fig E4, *B*), further arguing against the formation of a disulfide bond as the mechanism of increased p.W655C NLRC4 activation.

**Fig 6:**
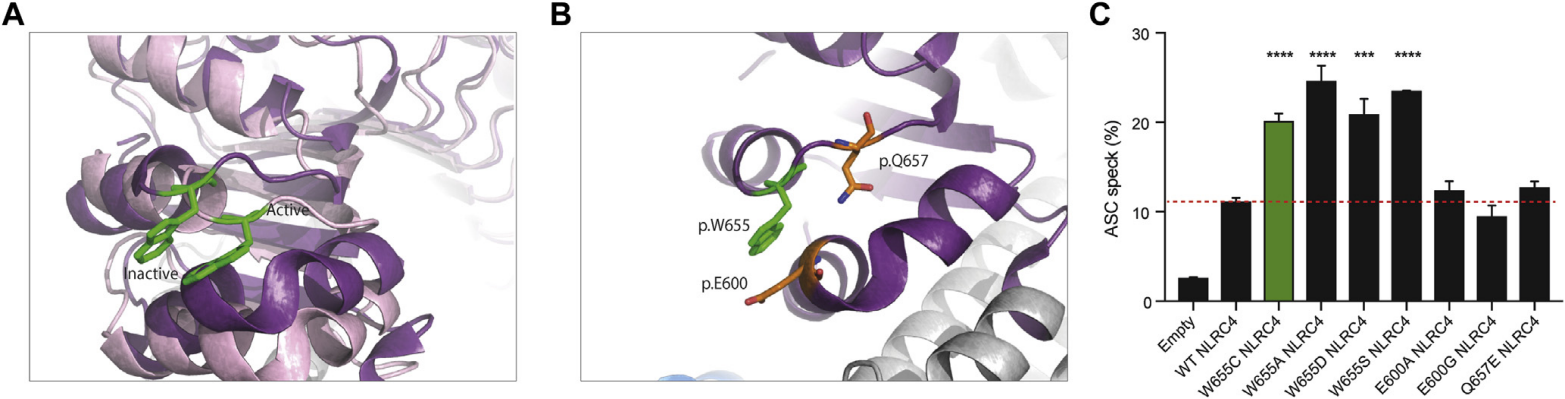
Assessment of local conformational changes in response to NLRC4 activation. **A,** Ribbon representation of the region surrounding p.W655 displaying conformational changes between autoinhibited (dark purple) and active (light purple) NLRC4. p.W655 NLRC4 is highlighted in green. **B,** Residues potentially interacting with p.W655 at rest and maintaining NLRC4 in an autoinhibited conformation. **C,** Flow cytometric ASC speck quantification of WT and p.W655C NLRC4, along with mutations created to explore potential local interactions. Data were pooled from 3 independent experiments. ***P < .001 and ****P < .0001.

In response to NLRC4 activation, p.W655 undergoes a small change in orientation (Fig 6, *A*). We investigated the possibility that p.W655 interacts with an amino acid in close proximity to keep NLRC4 in an autoinhibited conformation and whether substitution with cysteine results in loss of this interaction and subsequent activation (Fig 6, *B*). Substitution of the glutamic acid at p.600 to alanine or glycine to explore changes in polarity and size or the glutamine at p.657 to glutamic acid to explore changes in charge did not result in increased ASC speck formation (Fig 6, *C*). These findings suggest that disruption of local interactions when NLRC4 is in an autoinhibited conformation are unlikely to explain the increased activation in p.W655C NLRC4. Next, we asked whether increased NLRC4 activation was specific to the cysteine substitution at this residue. Tryptophan was mutated to alanine, aspartic acid, or serine to explore changes in size, charge, and polarity. Each of these mutations resulted in increased ASC speck formation when compared with WT NLRC4 (Fig 6, *C*), suggesting that p.W655 does not tolerate substitution and that changes are not specific to a cysteine at p.655.

### The LRR interface is important for inflammasome assembly

**Fig 7:**
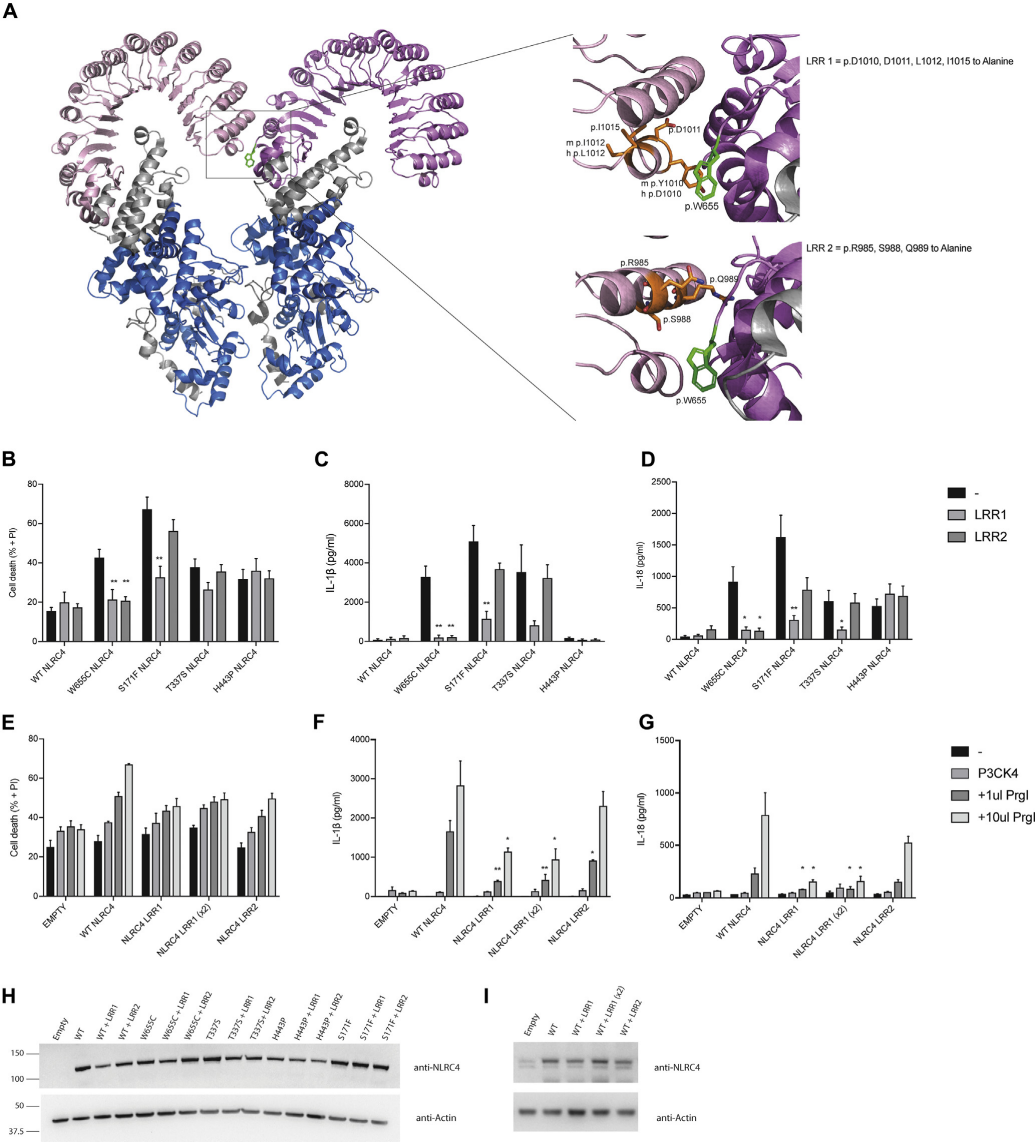
Assessment of p.W655 NLRC4 in oligomerized form. **A,** Ribbon representation of NLRC4 in the oligomerized state (PDB Code 6B5B^24^) with magnified inset areas highlighting 2 potential regions of interaction with p.W655 (LRR1 and LRR2). *NLRC4* KO THP-1 cells transduced with lentiviral constructs expressing WT or mutant NLRC4 combined with LRR1 or LRR2 mutations. **B-D,** Twenty-four hours after transduction, cells were primed with Pam3CSK4 for 24 hours, and then propidium iodine *(PI)* staining was assessed by using flow cytometry to quantify cell death (Fig 7, *B*), and IL-1β (Fig 7, *C*) and IL-18 (Fig 7, *D*) secretion were measured by ELISA. *NLRC4* KO THP-1 cells were transduced with 1 mL of WT *NLRC4*, LRR1 *NLRC4*, or LRR2 *NLRC4* virus or 2 mL of LRR1 *NLRC4* virus (LRR1 [×2]). **E-G,** After 24 hours, cells were infected with a retroviral PrgI construct and cell death (Fig 7, *E*), and IL-1β (Fig 7, *F*) and IL-18 (Fig 7, *G*) secretion assessed the following day. **H** and **I**, NLRC4 expression was assessed by using Western blot analysis. Data were pooled from at least 3 independent experiments. *P < .05 and **P < .01.

Next, we considered whether an LRR-LRR interface was important for the increased inflammasome activation seen with p.W655C. Several mutations to alanine were made in 2 separate α-helices to remove potential binding surfaces in the adjacent LRR of the activated NLRC4 oligomer (LRR1: p.D1010, p.D1011, p.L1012, and p.I1015; LRR2: p.R985, p.S988, and p.Q989; Fig 7, *A*). Cell death and cytokine responses of LRR1 and LRR2 NLRC4 transduced into *NLRC4* KO THP-1–Cas9 cells showed no difference when compared with WT NLRC4 (Fig 7, *B*). Combining p.W655C with either LRR1 or LRR2 reduced cell death and IL-1β and IL-18 secretion to levels comparable with WT NLRC4 (Fig 7, *B-D*). This suggests that the characteristics of the adjacent LRR and potential interactions at this interface are important for increased activation seen with p.W655C. Of note, addition of LRR1 mutations to a representative disease-causing mutation involving either the NBD, HD1, or WHD of NLRC4 resulted in significantly reduced cytokine secretion and cell death, with the exception of p.H443P NLRC4, implying that LRR1 might be important for NLRC4 oligomerization. On the other hand, combining LRR2 with known disease-causing mutations did not reduce inflammasome activation, suggesting that LRR2 is an interface that might only be engaged by the specific p.W655C NLRC4 mutation. This possibility was further explored through stimulation of WT, LRR1, or LRR2 NLRC4−expressing THP-1–Cas9 cells with the T3SS effector PrgI (Fig 7, *E-G*). Indeed, cells expressing LRR1 NLRC4 released significantly less IL-1β in response to PrgI compared with WT NLRC4 (Fig 7, *F*), even when increased amounts of LRR1 NLRC4 were expressed in the cells. We conclude that LRR1 is important for maximal physiologic oligomerization of the NLRC4 inflammasome complex.

## Discussion

We report the unfavorable clinical course of 2 children with MAS associated with the c.G1965C/p.W655C mutation in *NLRC4* and provide evidence of pathogenicity for this mutation in both a HEK293T overexpression model and genetically modified THP-1 cell models.

The clinical and biochemical evidence of MAS and gastrointestinal symptoms in our patients was similar to the original description of AIFEC.^6^, ^10^ Clinical symptoms and resistance to multiple immunomodulatory agents together with increased serum levels of free IL-18 prompted consideration of the diagnosis of AIFEC and a trial of IL-18BP in patient P1. However, severe end-organ damage after a prolonged disease course resulted in discontinuation of IL-18BP treatment and a change to palliative care. Although serum ferritin levels decreased in response to treatment with IL-18BP, effects cannot be adequately assessed after only 5 doses (Fig 1, *G*). Thus comparison with a recent case report of successful treatment of AIFEC with the IL-18BP Tadekinig is problematic.^34^ The duration of clinical disease and/or the severity of illness with organ damage before treatment initiation or differences in IL-18BP and free IL-18 might explain variable outcomes. Both patients with the p.W655C NLRC4 mutation succumbed to disease very early in life. Poor outcomes in the 2 reported patients with p.W655C NLRC4 mutations might reflect unfavorable genotype-phenotype correlations in patients with this form of NLRC4-AID.

The detection of *S paucimobilis* in the CSF of patient P2 raises the question of an immunodeficiency associated with p.W655C NLRC4. This gram-negative bacillus is generally considered to be of low virulence.^35^ A series of case reports of bacteremia with *S paucimobilis*, usually from intravenous administration of contaminated solutions in a health care setting, indicate that patients respond well to treatment, and only 1 pediatric and 1 adult case have been associated with mortality from this organism.^36^, ^37^ The possibility of an associated immune deficiency is raised by the severity of illness, as well as evidence of a poor IL-1β, IL-18, and cell death response of monocytes from patients with AIFEC to *S typhimurim* and *Pseudomonas aeruginosa* when compared with healthy control subjects.^10^ There has also been a report of activation of NLRC4 resulting in dampening of Toll-like receptor 5–induced antibody response to flagella, raising the possibility of an associated immunodeficiency.^38^ However, patient P2 cleared the organism with appropriate antimicrobial therapy as repeat CSF cultures were negative. Therefore it is difficult to conclude that the response to this flagellated organism was impaired. Alternatively, it might be that this infection with *S paucimobilis* triggered MAS. A further consideration is the possibility of sample contamination rather than a true infection.

This previously unknown c.G1965C/p.W655C *NLRC4* mutation causes increased inflammasome formation, cell death, and proinflammatory cytokine production in a caspase-1–dependent manner (Fig 5, *E-G*). The role of ASC in the response of NLRC4 to infection has been explored in murine gram-negative infection models, including *S typhimurium*, *P aeruginosa*, *Legionella pneumophila*, and *Shigella* species. In these disease models NLRC4 was required for both pyroptosis and cytokine production, but ASC was only required for cytokine responses.^39^, ^40^, ^41^, ^42^, ^43^ Here we show that in human cells ASC is required for IL-18 and possibly IL-1β production but not cell death. Therefore induction of cell death and cytokine production by caspase-1 might involve distinct pathways, with ASC required for one but not the other. Alternately, the level of caspase-1 activity required for cell death, and possibly IL-1β production, might be lower than that required for IL-18 production. NLRC4 might associate with caspase-1 independently of ASC because it contains a CARD domain. However, ASC may still be required for optimal inflammasome assembly and caspase-1 activation, and the absence of ASC might result in cell death without a maximal cytokine response.

Interestingly, p.H443P NLRC4 caused less ASC speck formation in the HEK293T system, as well as lower cytokine response, compared with other known disease-causing mutations in a THP-1 cell system. To date, p.H443P NLRC4 has been described in 1 Japanese family with familial cold autoinflammatory syndrome.^8^ No family member had MAS, which is consistent with a less severe clinical presentation.

This is the first report of a mutation in the LRR of NLRC4 that causes disease. By exchanging p.W655 for aspartic acid, alanine, or serine, we deduced that the size of tryptophan at p.W655 is potentially important for keeping NLRC4 in an autoinhibited conformation. Tryptophan is the largest amino acid, and substitution with smaller amino acids resulted in similarly increased ASC speck formation, regardless of changes to charge or polarity.

Furthermore, our data suggest that a structural change at position 655 creates a binding interface together with LRR domain residues p.R985, p.S988, and p.Q989 in the active oligomeric structure (Fig 7, *A*). Changes to the LRR adjacent to p.W655 disrupt this interface and abrogate increased inflammasome activation caused by the p.W655C mutation. The LRR1 interface (p.D1010, p.D1011, p.L1012, p.I1015) appears to be important for oligomerization of NLRC4, as the cytokine and cell death response of known mutations is abrogated when combined with LRR1, as is the response to PrgI.

Taken together, this study highlights the broad spectrum of clinical features and the severity of disease that can be seen in patients with NLRC4-AIDs. We model the first LRR mutation in NLRC4, p.W655C, to evaluate pathogenicity and show that the location of this residue is important in the mechanism of inflammasome assembly. Severe disease presentation and poor disease outcomes in both patients might reflect particularly unfavorable genotype-phenotype correlation for the p.W655C NLRC4 mutation in patients with this syndrome.

**Key messages**

• Two patients with p.W655C NLRC4 had fatal MAS and significantly increased serum IL-18 levels.
• *De novo* c.G1965C *NLRC4* mutation encoding p.W655C NLRC4 is the first mutation reported in the LRR domain of NLRC4 to cause disease.
• An LRR-LRR interface is important for NLRC4 inflammasome activation by NLRC4-AID mutations and the T3SS effector PrgI.

## Figures and Tables

**Table E1:** Primers

Primer	Sequence (5′ → 3)
Amplification	
Lenti_blast _F	GCGGATCCAGCAGTTACTAGTTTAAAAGC
Lenti_blast_R	CACAGGACCGGTTCTAGAGCGCTGCCACCATGAATTTCATAAAGGACAATAG
CRISPR sgRNA	
Exon 2_1	TCCCTCAGCTGCTCCACGCGGTGA
Exon 2_1	AAACTCACCGCGTGGAGCAGCTGA
Sequencing	
NLRC4_ex3_F	TGA ATA GCC AGG GGC TTT GAG
NLRC4_ex3_R	ACT GGT CAG AGG AAG CCA TG
NLRC4_ex4_F	AGT AGA GAC AGG GTT TCA CTG
NLRC4_ex4_R	TAT CAA AGG CCA CTG CCA GG
NLRC4_ex5_Fa	AGC TCA GAA CTG GGA AAG GAC
NLRC4_ex5_Ra	TGC TTC CTG ATT GTG CCA GG
NLRC4_ex5_Fa1	AAG TGC AAG GCT CTG ACC AAG
NLRC4_ex5_Ra1	TCA TGA GAT TCC TCA AGC ACC TG
NLRC4_ex5_Fa2	AGT TTG GTG CCC TGA CTG C
NLRC4_ex5_Ra2	TGC TGA GTC TTC GTC CTG C
NLRC4_ex5_Fa3	ACT GCA GGA TGT GTC CAG C
NLRC4_ex5_Ra3	TGA AGT CCA GGG CAC TTG C
NLRC4_ex5_Fa4	AGT ACA TCC AAA TCA GCC CTG
NLRC4_ex5_Ra4	TTG CAG CCT GAG GCT TGT G
NLRC4_ex5_Fa5	AAC CTA CAT TCC CAG CAG GG
NLRC4_ex5_Ra5	ACC TTG CAG GCT GTA ACC C
NLRC4_ex6_F	ACA GTG AAG TAT AAT GCC CAT CTC
NLRC4_ex6_R	ACA TCC TTG CCT TGT GCA GAC
NLRC4_ex7_F	AGT CGC TGC AGG AAG AAC ATG
NLRC4_ex7_R	TGT GTG GCA AGT TTC AAG CAC C
NLRC4_ex8_F	TAC ACC TAC ACC AGA GCC TTT G
NLRC4_ex8_R	AGG AGG GTG CAT CTA GTA AGG
NLRC4_ex9_F	AGA TTG AGT AGG CTG CAG AGC
NLRC4_ex9_R	AGA ATC TGA AGG AAG GCC AGT G
NLRC4_ex10_F	TCT GAG GCA AAG CAC TGG TTC
NLRC4_ex10_R	TCC TAG TAG GAG AGC TAA GAC AC
Forward	CAAAGTGTGAAAAACACCACTGAGC
Forward	GTGTTTTTTGACTTTAGTAC
Forward	AGGACTTGAATGGACAAAGTC
Mutagenesis	
p.W655C	TATCTTTGTTCTTCAACTGCAAGCAGGAATTCAGGACTC
p.W655C	GAGTCCTGAATTCCTGCTTGCAGTTGAAGAACAAAGATA
p.T177A	TCGCTGCAGCAGAGCGGACTTGCCTTTGC
p.T177A	GCAAAGGCAAGTCCGCTCTGCTGCAGCGA
p.S171F	TTGCCTTTGCCAAATTCCCCTTCAATGATGCAGGGG
p.S171F	CCCCTGCATCATTGAAGGGGAATTTGGCAAAGGCAA
p.W655A	GAGTCCTGAATTCCTGCTTCGCGTTGAAGAACAAAGATACAG
p.W655A	CTGTATCTTTGTTCTTCAACGCGAAGCAGGAATTCAGGACTC
p.W655S	AGTCCTGAATTCCTGCTTCGAGTTGAAGAACAAAGATAC
p.W655S	GTATCTTTGTTCTTCAACTCGAAGCAGGAATTCAGGACT
p.S533A	TTTTCACACTTTGCAAAGCTTCCTGTCTCCAGAGAGG
p.S533A	CCTCTCTGGAGACAGGAAGCTTTGCAAAGTGTGAAAA
p.E600A	ACTTGCACAATTGGGCAAATGTGCAAAGAAGTCAAATAAGTAATCG
p.E600A	CGATTACTTATTTGACTTCTTTGCACATTTGCCCAATTGTGCAAGT
p.E600G	ACTTGCACAATTGGGCAAATGTCCAAAGAAGTCAAATAAGTAATCG
p.E600G	CGATTACTTATTTGACTTCTTTGGACATTTGCCCAATTGTGCAAGT
LRR1	CAGTTACTAGTTTAAAAGCACCTGTAGCAACACTGGCAGCAGCATCATCAAATTGCCACCCAACAAGCC
LRR1	GGCTTGTTGGGTGGCAATTTGATGATGCTGCTGCCAGTGTTGCTACAGGTGCTTTTAAACTAGTAACTG
LRR2	CTTCTTGCAGAAAAGTTAACTTGGATAACACTGCGGCAAGTTTTGCGACTAATGCTGGATCAGGTAGAAATTCTTTAG
LRR2	CTAAAGAATTTCTACCTGATCCAGCATTAGTCGCAAAACTTGCCGCAGTGTTATCCAAGTTAACTTTTCTGCAAGAAG
p.V341A	TTGCACAAGTGATGACCGCAAAGAGAGGGGTCTTC
p.V341A	GAAGACCCCTCTCTTTGCGGTCATCACTTGTGCAA
p.T337S	TGAGGAATCTCATGAAGTCCCCTCTCTTTGTGGTC
p.T337S	GACCACAAAGAGAGGGGACTTCATGAGATTCCTCA
p.Q657A	TTTGTTCTTCAACTGGAAGGCGGAATTCAGGACTCTGGAG
p.Q657A	CTCCAGAGTCCTGAATTCCGCCTTCCAGTTGAAGAACAAA

*sgRNA*, Single guide RNA.

**Table E2:** Immunologic workup of patient P2

Test	Result	Reference range
Immunoglobulins		
IgG (g/L)	23.5	4.5-9.5
IgA (g/L)	0.18	0.2-1
IgM (g/L)	1.18	0.2-1.46
IgE (IU/mL)	6140	<200
Complement		
C3 (g/L)	1.79	0.88-2.52
C4 (g/L)	0.12	0.12-0.72
Lymphocyte subsets		
CD3^+^ (cells/μL)	3560.6	2542-4933
CD3^+^CD4^+^ (cells/μL)	1900.4	1573-2949
CD3^+^CD8^+^ (cells/μL)	1413.9	656-1432
CD19^+^ (cells/μL)	540.8	733-1338
NK cells (cells/μL)	211.83	186-724

*NK*, Natural killer.
